# Counting what counts: a systematic scoping review of instruments used in primary healthcare services to measure the wellbeing of Indigenous children and youth

**DOI:** 10.1186/s12875-023-02001-z

**Published:** 2023-02-17

**Authors:** Vicki Saunders, Janya McCalman, Sena Tsey, Deborah Askew, Sandy Campbell, Crystal Jongen, Candace Angelo, Geoff Spurling, Yvonne Cadet-James

**Affiliations:** 1 Jawun Research Centre, Central Queensland University, Cnr Shields and Aplin St, Cairns, QLD 4870 Australia; 2 General Practice Clinical Unit and School of Public Health, Faculty of Medicine, University of Queensland, Level 2, Public Health Building, 288 Herston Road, Brisbane, QLD 4006 Australia; 3 Molly Wardaguga Research Centre, Charles Darwin University, Level 11, 410 Ann St, Brisbane, QLD 4000 Australia; 4 Aboriginal and Torres Strait Islander Public Health, Sydney School of Public Health, Faculty of Medicine and Health, University of Sydney, Edward Ford Building, Fisher Road, Sydney, NSW 2006 Australia; 5 Yvonne Cadet-James, Apunipima Cape York Health Council, 186 McCoombe St, Cairns, QLD 4870 Australia

**Keywords:** Screening, Assessment, Measure, Wellness, Mental health, Adolescence

## Abstract

**Background:**

Primary healthcare services have principal responsibility for providing child and youth wellbeing and mental health services, but have lacked appropriate measurement instruments to assess the wellbeing of Indigenous children and youth or to evaluate the effectiveness of programs and services designed to meet their needs. This review assesses the availability and characteristics of measurement instruments that have been applied in primary healthcare services in Canada, Australia, New Zealand and the United States (CANZUS countries) to assess the wellbeing of Indigenous children and youth.

**Methods:**

Fifteen databases and 12 websites were searched in December 2017 and again in October 2021. Pre-defined search terms pertained to Indigenous children and youth, CANZUS country names, and wellbeing or mental health measures. PRISMA guidelines were followed, with eligibility criteria guiding screening of titles and abstracts, and selected full-text papers. Results are presented based on the characteristics of documented measurement instruments assessed according to five desirability criteria: development for Indigenous youth populations, adherence to relational strength-based constructs, administration by child and or youth self-report, reliability and validity, and usefulness for identifying wellbeing or risk levels.

**Results:**

Twenty-one publications were found that described the development and or use by primary healthcare services of 14 measurement instruments, employed across 30 applications. Four of the 14 measurement instruments were developed specifically for Indigenous youth populations, four focused solely on strength-based wellbeing concepts but none included all Indigenous wellbeing domains.

**Conclusion:**

There is a diversity of measurement instruments available, but few fit our desirability criteria. Although it is possible that we missed relevant papers and reports, this review clearly supports the need for further research to develop, refine or adapt instruments cross-culturally to measure the wellbeing of Indigenous children and youth.

**Supplementary Information:**

The online version contains supplementary material available at 10.1186/s12875-023-02001-z.

## Background

The World Health Organization (WHO) recommends that the primary responsibility for child and youth wellbeing and mental health services lies with primary health care (rather than specialist) level, close to communities and with young peoples’ active engagement in care [1]. Primary healthcare services provide and facilitate access to a spectrum of programs and services from mental health promotion, screening and early identification and diagnosis to treatment of mental disorders [2]. But how is wellbeing for Indigenous youth conceptualised? By what process are the wellbeing measures decided? And who makes those decisions? These are critical questions being raised within the primary healthcare service delivery sectors in the settler colonised countries of Canada, Australia, New Zealand and the United States (CANZUS countries). These questions speak to the values that underpin what counts in assessments of the wellbeing of individual Indigenous children or youth, and evaluations of services and programs for and with Indigenous children and youth. In this review, wellbeing is defined as a relational phenomenon encompassing individual, collective, cultural and spiritual domains.

It is well established that the school-aged years of childhood (defined in this review as 5–11 years old) and youth (defined as those aged 12–18 years old) are critical for fostering wellbeing, preventing mental illness and intervening early to improve life outcomes, since 75% mental illnesses globally starts by age 25 years [3]. In Australia, for example, a national survey in 2021 found that 39.2% of Indigenous youth (aged 15–19 years) reported mental health as one of their top three personal issues of concern [4]. The found fewer Indigenous Australian youth reported that they were ‘very happy’ with their lives in 2021 (42%) than 2020 (45%). A higher proportion also reported unfair treatment in 2021 (47%) than 2020 (39%), and a similar proportion reported high levels of stress in the previous 4 weeks in 2021 (43%) and 2020 (44%) [4, 5]. Yet in Australia, our analysis of audits from 114 Indigenous PHC found only 27% of clients were screened for social and emotional wellbeing (SEWB). Of the 13% for whom concerns were identified, further action was taken for only 75% and of those for whom action was taken, there was internal follow up within a month for only 55%, and follow up for those referred to other services within six months of only 51% [6].

Furthermore, another survey found that 77% of Indigenous Australian young people (18–24 years) with high levels of distress reported that they did not even see a primary healthcare professional [7]. Hence, the needs of Indigenous children and youth for mental health care services are often unmet [8]. One barrier to routine wellbeing screening of Indigenous children and youth is a lack of appropriate measurement instruments for opportunistic screening or program evaluation by primary healthcare services [9].

This review identifies and describes the evidence (January 1989 to October 2021) about the availability and characteristics of wellbeing and mental health measurement instruments used within primary healthcare settings in CANZUS countries to screen Indigenous children and youth (5–18 years) or measure intervention effects. It forms part of a broader program of work with Indigenous Australian primary healthcare services to improve systems and services that are tasked to promote the wellbeing of Indigenous children and youth [10–12]. It was conducted by leaders of a SEWB research program that forms part of the Australian Centre of Research Excellence for STRengthening InDigenous healthcare Equity (CRE-STRIDE). The SEWB program of CRE-STRIDE aims to co-design, develop, apply and evaluate continuous quality improvement approaches to support the SEWB of Indigenous young people in Australia. The authors are current or former program leaders, the program’s post-doctoral researcher, research assistants and a general practitioner. Four co-authors identify as Indigenous to Australia, and five identify as Australians no longer indigenous to place. The authors acknowledge the significance of Country, place and land in the work we do.

In making decisions about the use of instruments to measure Indigenous youth wellbeing, primary healthcare services are faced with a complex choice [9]. The international evidence suggests that there are five interrelated characteristics of measurement instruments that can be considered, with appropriateness influenced by the local need and Indigenous youth population group [9, 13]. These characteristics are 1) development specifically for Indigenous youth wellbeing, 2) incorporation of relational and strengths-based concepts, 3) self-administration by Indigenous children or youth, 4) reliability and validity, and 5) utility for measuring wellbeing or risk levels.

Indigenous youth wellbeing is often measured using instruments designed for non-Indigenous populations [9, 13]. Whilst the use of measurement tools validated with non-Indigenous populations allows comparison with other data sets and populations, they do not measure wellbeing as it is conceptualised in Indigenous frameworks, using appropriate language to describe wellbeing, or framing norms of emotional expression and behaviour that are appropriate in Indigenous situations [9, 14]. There are, however, instruments developed for wellbeing measurement that are designed for specific Indigenous youth population groups and that potentially can be modified for other Indigenous youth populations [15].

The wellbeing of Indigenous youth, has often been measured using Western concepts understand wellbeing primarily at an individual level as a positive rather than neutral state [16]. In contrast, Indigenous views of wellbeing encompass relational and strengths-based concepts of wellbeing at multiple levels [13]. These encompass individual (physical, mental and emotional), collective (family, kinship, tribal and community), cultural (language, cultural knowledge and cultural practice) and spiritual (spirituality, ancestors and land) domains [13]. For example, Indigenous Australians use the term SEWB to refer to connections of the self to and interconnections between physical health, mental/emotional health, kinship, community, culture, Country, and spirituality and or ancestors [17]. Inuit, Métis, and First Nations Canadians understand the wellness of a strong and healthy person as derived from connections with the distinct waters, lands and natural world. The well person lives in relational harmony with others, with community and nation, as well as with the temporal and spirit worlds [18, 19]. The Aotearoa New Zealand Māori *Whare tapa whā* model of health is based on four interconnected elements of life: the physical, emotional, mental, and spiritual [20]. American Indian and Alaska Native peoples also focus on a wholistic and collective view of wellness as physical and mental, cultural and spiritual, and in which lands and place play a prominent role [21]. Some instruments incorporate relational and strengths-based constructs, whereas others aim to identify pathological states.

Within primary healthcare services, measurement tools are generally administered by clinicians or researchers to children and youth. These administrators interpret the responses of child or youth experiences or perceptions of wellbeing, including their emotions, motivations, spirituality, relationships and cultural connection [22]. The principles of self-determination have been identified as important to Indigenous wellbeing, including being protective against Indigenous youth suicide [13, 23]. Indigenous children and youth-reported outcomes provide direct responses from the perspectives and experiences of the child or youth [8, 24].

Primary healthcare services also seek measurement instruments that are reliable and have been validated. The reliability (whether the results can be reproduced under the same conditions) and validity (extent to which the results truly represent what they are supposed to measure) of measurement instruments enables primary healthcare services to have confidence in the results of measures [9]. There are instruments that are reliable and valid for specific Indigenous youth populations, some that are reliable and valid for general populations, and some that have been found not to be reliable and or valid for use across Indigenous youth populations.

This review updates a 2013 review by Williamson et al. [9] that included fifty-four studies from CANZUS nations, published from 1998 to 2008 across any setting (primary healthcare, school, child protection, juvenile justice etc.), and provides a deep dive description and critical evaluation of the measurement instruments used specifically in primary healthcare settings. Williamson et al. [9] found that seventy-nine mental health instruments were used across sectors, but only 11 (14%) instruments had been specifically developed for use with Indigenous people or validated for the relevant Indigenous population [9]. More recent reviews have described or evaluated interventions to enhance the wellbeing of Indigenous people generally [8, 25–29] or youth more specifically [30–32]. There are also recent reviews that describe or evaluate measures of Indigenous wellbeing for adults [33, 34]. To the best of our knowledge however, this is the only recent review focused on measures of wellbeing used in primary healthcare services to measure the wellbeing of Indigenous children and youth.

Hence the aim of this review was to determine the evidence about what instruments have been used in CANZUS primary healthcare services to measure the wellbeing of Indigenous children and youth (5–18 years), and to what extent: 1) are they developed for Indigenous children or youth, 2) do they incorporate relational and strengths-based concepts of wellbeing, 3) are they administered through child and or youth self-report; 4) are they valid and reliable, and 5) are the useful for measuring wellbeing or risk levels?

## Methods

A protocol was written to guide this systematic scoping review to identify relevant publications that provided evidence about measures used in CANZUS primary healthcare services to assess the wellbeing of Indigenous children and youth or the effects of programs for Indigenous child and youth wellbeing. The protocol was not registered with an a priori review service but is available from the corresponding author. Studies were included if they reported the development or use of measures. Heeding the call from Indigenous scholars for strengths-based rather than deficit-focussed research and practice [35], we used mainly strength-based terms in our search.

### Search strategy

**Table:** 

**Electronic database search**: First, an exploratory search was carried out in the following databases and selected references were downloaded: Scopus / Elsevier, PubMed Clinical Queries and the Learning Ground Indigenous Education Research / ACER databases
A comprehensive search was then completed in: Medline (including Epub Ahead of Print, In-Process & Other Non-Indexed Citations) / Ovid; Embase / Ovid; PsycINFO / Ovid; EBM Reviews—Cochrane Database of Systematic Reviews / Ovid; CINAHL / Ebsco; Global Health/ Ovid; ATSIHealth /Informit; APAIS-ATSIS / Informit; AIATSIS: Indigenous Studies Bibliography/ Informit; FAMILY-ATSIS / Informit; ERIC / Proquest; A + Education / Informit; PAIS / Proquest; Sociological Abstracts / Proquest. A search in The Campbell Library database did not retrieve any relevant studies. Searches were completed on 6, 8–10, 12–18 December 2017. Searches were updated, completed on 21 October 2021
Search strategy: The databases were searched with the terms below (and their corresponding subject headings in each database where specialised thesauri existed):
1. Indigenous OR Aborigin* OR “Torres Strait Island”* OR Inuit OR Māori OR Iwi OR Tangata Whenua OR “First Nation”* OR Metis OR “Native American”* OR “American Indian”* OR “Native Hawaiian” OR tribal
2. adolescen* OR youth* OR young people OR young adult* OR child* OR teen* OR juvenile*
3. wellbeing OR mental health OR wellness OR healing OR *stress
4. screen* or assess* or path* OR model OR manage* OR refer* OR tool OR measure OR indicator
5. Australia OR Canada OR USA OR New Zealand
6. 4 OR 5
7. 1 AND 2 AND 3 AND 6 AND 7
**Websites manually** searched included:
◾Google Scholar and Google
◾ **Australia:** Indigenous HealthInfoNet; Closing the Gap Clearinghouse
◾**Canada:** The National Collaborating Centre for Aboriginal Health; (National Aboriginal Health Organisation was closed); Health Council of Canada: Aboriginal Health
◾ **New Zealand:** Māori Health; Whakauae: Research for Māori Health and Development; MAI: A New Zealand Journal for Māori Health and Development
◾**USA:** American Indian Health; National Indian Health Board; Centres for American and Alaska Native Health
The grey literature search terms were:
1. Indigenous OR First Nation* OR Inuit OR Metis OR Aborigin* OR Torres Strait Island* OR Māori OR Iwi OR Tangata Whenua OR Native American* OR Native Alaskan* OR Native Hawaiian* OR Indian OR tribal AND
2. Wellbeing OR mental health AND
Screening OR assessment OR measurement

We acknowledge that the use of terms to describe sovereign Indigenous peoples is contentious. We included a broad range of search terms in recognition of the potential use of these terms in publications going back to 1989.

### Inclusion and exclusion criteria

**Fig. 1:**
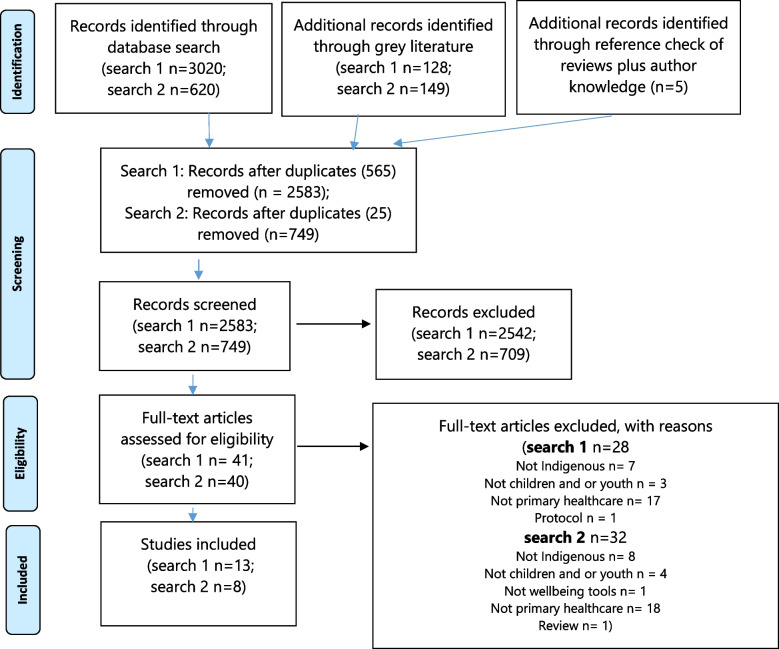
PRISMA flow diagram of search strategy

An initial literature search was conducted of peer-reviewed and grey literature published in English from January 1989 to December 2017. PhD theses were excluded because there is evidence that when systematic reviews include theses, they mostly had little impact on results [36]. Other reviews and protocols were also excluded. The start date was taken to coincide with the holistic view of Indigenous health identified in Australia by the National Aboriginal Health Strategy Working Party (NAHS, 1989). Although Australian, this seminal strategy has relevance for other CANZUS nations in that it acknowledged diversity across Indigenous peoples and the multitude of deeply harmful effects of colonisation, and introduced fundamental changes based on a preventive healthcare approach. A second search was conducted in October 2021 to update the review from 2018–2021 (Fig. 1). Publications were included which met the following criteria:

1. The study is from Australia, Canada, NZ or USA; and
2. The publication includes Indigenous children and or youth from Australia, Canada, NZ or USA; and
3. The children and or youth are aged 5–18 years; and
4. The study describes or evaluates wellbeing or mental health screening or measurement tools used with Indigenous children or youth; and
5. The study setting is a primary healthcare service.

Each eligible full text publication was screened by two blinded assessors (one Indigenous, one non-Indigenous), with each publication being rated yes, no, or unclear against each of the screening criteria. Differences in assessments, or ratings of “unclear” were resolved between the two assessors by consensus. The publication was included only if it was scored with a “yes” response across all criteria.

### Data extraction & synthesis

Data were extracted from the full texts of studies (by CJ, ST and JM) into a table (supplementary table). The domains extracted for each publication included: authorship, year and type; country and target group; sample size and measurement setting; measurement type and study design; outcome measures; reported outcomes; and study quality.

### Assessing the characteristics of measurement instruments

The measurement instruments were then assessed against five desirability criteria that were informed by the international literature. The review of Indigenous wellbeing by McClintock, King [13] which was authored by an international group of Indigenous authors was particularly influential. The criteria were: development for Indigenous youth populations, incorporating relational strength-based constructs, administration by child and or youth self-report, reliability and validity, and utility for identifying wellbeing or risk level outcomes.

#### Development for indigenous youth populations

The benefits of specifically-developed Indigenous measurement tools were considered likely to better encompass Indigenous youth views of their own wellbeing.

#### Constructs of indigenous wellbeing

Scales that predominantly measured mental distress or ill-being (e.g., depression, suicide ideation or risk, impulsivity and anxiety) provide little meaningful insight into wellbeing which is a positively loaded term that implies more than the absence of pathology or illness [37]. A focus of the measure on relational and strengths-based constructs was therefore considered desirable.*Administration by child or youth self-report:* Studies with child or youth-reported outcomes were considered important because the ratings by the Indigenous children and or youth themselves were prioritised, without interpretation of their response by a parent, carer or clinician [22, 38]. The reporting on wellbeing measures by children’s and youth directly means that they speak for themselves, rather than being spoken for. Other studies have shown that service providers perceptions about what is needed for youth wellbeing differ from the views of young people themselves [39].

#### Reliability and validity

In this review, we identified the extent to which the measure was considered in the primary study to be reliable under the same conditions over time and valid for the Indigenous youth population to whom the instrument was administered (see supplementary table 1).

#### Useful for identifying wellbeing or risk level outcomes

The value of wellbeing measurement tools in primary healthcare services is either to identify changes in child and youth wellbeing over time or to identify wellbeing risks and take action and follow up [6]. The publications were assessed to determine whether they reported the outcomes of measurement for these purposes.

## Results

A total of 21 publications were identified that evaluated or described wellbeing measures used in primary healthcare services for Indigenous children and youth [38, 40–55]. Nine (43%) publications came from Australia, six (29%) from Canada, five (24%) from Aotearoa New Zealand, and one (5%) from the United States. The 21 publications represented nine studies and described the development and or use of 14 measurement tools, employed across 30 applications. The publications are summarised in Table 1.

**Table 1:** Summary of the publications

Studies Author, year	Country	Measures reported	Target population of Indigenous children and youth		Study purpose			
			**Universal**	**Specific mental health concern**	**Describe development of Indigenous-specific tool**	**Trial Western measure with an Indigenous population**	**Identify wellbeing risks**	**Test to identify wellbeing improvement over time**
**Clark 2014 ** [40]	NZ	Children’s Global Assessment Scale (C-GAS); Strengths and Difficulties Questionnaire (SDQ); Substance Abuse Choices Scale (SACS)	✓	✓				✓
**Coffin 2019 ** [47]	Aus	SDQ	✓			✓		
**Goodyear-Smith 2016 ** [55]**; 2017 ** [54]**; Thabrew 2019 ** [56]	NZ	YouthCHAT; Alcohol, Smoking and Substance Involvement Screening Test (ASSIST); Generalised Anxiety Disorder 7 (GAD-7); Patient Health Questionnaire 9 (PHQ-9); SACS	✓		✓	✓		✓
**Harriss 2018 ** [48]	Aus	Adapted Patient Health Questionnaire 9 (aPHQ-9)	✓			✓		✓
**Mitchell 2011 ** [41]	USA	Kessler Distress Scale (K6 +)	✓			✓		
**Sabbioni 2018 ** [53]	Aus	Outcome Rating Scale (ORS); Session Rating Scale (SRS)	✓	✓		✓		✓
**Thomas 2010 ** [42]**; Gorman 2021 ** [57]	Aus	Strong Souls; Westerman Aboriginal Symptoms Checklist- Youth (WASC-Y); K6 +	✓		✓			
**Williamson 2010 ** [43]**, 2014 ** [49]**, 2016 ** [44]	Aus	SDQ	✓			✓		✓
**Williamson 2018 ** [50]	Aus	ED presentations and hospital admissions		✓			✓	
**Young 2011, 2015 ** [45]**, 2015a ** [51, 58]**, 2016 ** [46]**, 2016a ** [52, 59]**, Usuba 2019 ** [60]	Canada	Aboriginal Children’s Health and Well-Being Measure (ACHWM)	✓		✓			✓

### Target population

Eighteen of the 21 publications (86%) targeted all Indigenous children and youth clients of that service (i.e. a universal approach), and three (14%) targeted Indigenous children and youth who had already reported mental health concerns [40, 51, 54] (Table 1).

### Study purpose

Eight publications documented the development of three measurement instruments to measure the wellbeing of Indigenous youth (Table 1) [38, 43, 46, 47, 52, 53, 55, 56]. The ACHWM was developed in Canada, the Strong Souls measure in Australia, and a Maori language version of the YouthCHAT in New Zealand. As well, the Westerman WASC-Y was included as a comparator Indigenous-developed measurement instrument in one publication [43], but its development was not described.

Six publications described assessments of appropriateness or trials of the validity of standardised Western measures in an Indigenous youth population. Five of these were from Australia [45, 48–50, 54]. The measures included the Outcome Rating Scale (ORS), Session Rating Scale (SRS), Strengths and Difficulties Questionnaire (SDQ), and adapted Patient Health Questionnaire (PHQ-9). The other publication was from the United States, [42] and focussed on the Kessler-6 (K-6) instrument.

Two Australian publications described the use of measurement instruments to identify risks to Indigenous youth wellbeing [45, 51]. The measurement indicators were the SDQ and mental health-related emergency department (ED) presentations and hospitalisations.

Five publications documented measures that were useful for measuring change over time in youth wellbeing outcomes [40, 49, 54, 60, 61]. Two studies were from Australia [49, 54] and measured service level results using the SRS and CRS; and youth wellbeing improvements using the a-PHQ-9. The outcomes are described in the next section. Two studies were from New Zealand [40, 60] and evaluated a referral-based free counseling support service for youth with mild to moderate mental health problems using the C-GAS, SDQ and SACS, and YouthCHAT to evaluate the mental health issues of youth with long-term physical conditions. Finally, from Canada, one study [61] used the ACHWM to evaluate an outdoor activity leadership experience.

### Characteristics of measures

Fourteen measurement instruments were documented in the included publications. Their characteristics are outlined in Table 2.

**Table 2:** Characteristics of measurement instruments

Measurement instrument	Country	Developed for Indigenous children and or youth	Relational strength-based constructs	Administered through self-report	Valid and reliable for Indigenous sample	Useful for identifying wellbeing or risk level
Aboriginal Children’s Health and Well-Being Measure (ACHWM)	Canada	√	√	√	√	√
Alcohol, Smoking and Substance Involvement Screening Test (ASSIST) for smoking	NZ	X	X	X	X	X
Children’s Global Assessment Scale (C-GAS)	NZ	X	√	X	√	√
Hospital admissions with a primary mental health diagnosis	Aus	X	X	X	√	√
Generalised Anxiety Disorder 7 (GAD-7)	NZ	X	X	X	X	√
Kessler Distress Scale abridged version (K6 +)	Aus, USA	X	X	X	√	√
Outcome Rating Scale (ORS)	Aus	X	√	√	√	√
Patient Health Questionnaire 9 (PHQ-9 and a-PHQ-9)^1^	Aus NZ	X	X	X	√	√
Session Rating Scale (SRS)	Aus	X	√	√	√	√
Strengths and Difficulties (SDQ)	Aus NZ	X	~	X	√	√
Strong Souls	Aus	√	~	√	X	X
Substance Abuse Choices Scale (SACS)	NZ	X	X	X	X	√
Westerman Aboriginal Symptoms Checklist- Youth (WASC-Y)	Aus	√	X	√	√	√
YouthCHAT	NZ	√	~	√	X	√

The a-PHQ 9 was adapted for use with an Australian Indigenous community [58]. The symbol √ denotes a clear description of this characteristic in the publication. The symbol ~ means that the characteristic was alluded to but not explicitly described. The symbol X means that the characteristic was not present

#### Indigenous-specific tools

Three measurement instruments were Indigenous-specific tools developed to measure Indigenous youth wellbeing (Table 1). The ACHWM was developed in Canada, the Strong Souls measure in Australia, and the Westerman WASC-Y was included as a comparator Indigenous-developed measurement instrument in one publication [43] (Table 2).

The Aboriginal Children’s Health and Well-Being Measure (ACHWM) was developed with and for children and youth (aged 8–18 years) from the from the Wikwemikong Unceded Indian Reserve in Canada. The tool includes positive wellbeing measures based on children’s and other community members’ understanding and perspectives on wellbeing. The ACHWM is a self-report survey that produces a score in four quadrants (mental, physical, emotional, and spiritual). Strategies such as using a tablet device with voice-to-text software were used to enhance the accessibility of the data being collected, allowing for greater participation. Having been collaboratively designed, studies have reported that Aboriginal children can interpret ACHWM questions consistently and accurately, making it reliable over time [56]. There is also evidence of its validity [46], appropriateness [57] internal consistency and test–retest validity [53], specificity, negative predictive value, and sensitivity [47]. The ACHWM was used to evaluate an outdoor activity leadership experience, with significant change in the well-being of the Indigenous youth participants over a short 10-day canoe excursion reported [61].

Strong Souls was developed with Indigenous youth in the Northern Territory of Australia. It is a 25-item survey which includes questions about both strengths and challenges to wellbeing. Thomas, Cairney [43] described its development and piloting alongside another Indigenous Australian tool, the Westerman Aboriginal Symptoms Checklist—Youth (WASC-Y). Later testing of the psychometric properties of Strong Souls with Indigenous youth in other parts of Australia, however, found that further modifications were required before the instrument could be considered a reliable measure for use with Indigenous youth [62]. The WASC-Y was Indigenous-developed in Western Australia and is validated with Indigenous Australian youth [49] but it is not freely available.

YouthCHAT is an electronic, self-report tool developed in New Zealand from eCHAT, a similar survey designed for adults that provides scores on 13 different domains. The YouthCHAT was designed for assessing the wellbeing of vulnerable youth, with a Maori language (Te Reo) version available. It includes questions about both strengths and challenges to wellbeing and enables the user to indicate the areas in which they would most like help. This information is immediately provided to the health provider, thus helping to streamline and tailor health assistance strategies. It also encourages users to have input into their management plans and arguably increases the likelihood of successful intervention. The acceptability and utility of YouthCHAT with Maori youth in New Zealand have been established [55, 56, 60] but YouthCHAT has not been independently tested for validity and reliability with Maori youth.

#### Western tools with relational strengths-based constructs

In addition to the Indigenous-developed tools, we found documentation of two measurement instruments that incorporated a relational and strengths-based approach that was somewhat consistent with Indigenous understandings of wellbeing. They were the session rating scale and outcome rating scale, both used in an Australian evaluation of a mental health service to determine service-level effectiveness [54].

The Session Rating Scale (SRS) is designed as a youth self-administered tool that includes four strengths-based questions relating to relationships, goals, topics and global rating. It requires youth participants to place a mark on a continuous scale, indicating their perception of their own rating against that domain. Sabbioni, Feehan [54] trialled the Session Rating Scale (SRS) and Outcome Rating Scale (ORS) with Indigenous Australian youth clients of Youthlink—a state-wide mental health service program for young people in Western Australia. The SRS and ORS have not yet been cross-culturally validated, but according to Sabbioni [54], were deemed appropriate for use by Indigenous youth. The tools are simple and brief, and successful in capturing clients’ perspectives about their treatment progress and the client’s perception of the therapeutic bond with the practitioner; hence they represent a valuable alternative approach to standardised measures. Sabbioni, Feehan [54] found pre-post improvements in youth-administered ORS scores across the treatment period at YouthLink, with 65% of clients improving, but 25% showed no change and 10% deteriorated. The Outcome Rating Scale (ORS) includes four main areas of inquiry capturing clients’ perspectives about the therapeutic bond: personal, relational, social and global. This scale is also self-administered by the participant. The final SRS correlated significantly with final ORS, suggesting an association between therapeutic alliance and treatment outcome, although the difference lay just outside of statistical significance.

The Strengths and Difficulties Questionnaire (SDQ) was reported in three included studies. The SDQ measures the strengths and difficulties of children including emotional symptoms, peer problems, hyperactivity inattention, conduct problems and prosocial behaviours. According to Williamson et. al. [44], Indigenous Australian youth, parents and Indigenous staff of four Aboriginal Community Controlled Health Organisations in New South Wales reported that the SDQ was acceptable for use with youth. However, changes were needed to the wording of some questions and the response scale to improve cultural appropriateness and clarity. Problems were also noted with the fit and validity in the peer relationships subscale and the authors recommended focusing instead on the SDQ total difficulties score [45, 63]. Administration of the SDQ to carers of Indigenous Australian youth clients of four ACCHOs found that 72% were not at high risk for emotional or behavioural problems [45]. After adjusting for demographic and health characteristics, the factors that were associated with good mental health were having a carer who was not highly psychologically distressed; not suffering from frequent chest, gastrointestinal or skin infections; and eating two or more servings of vegetables per day. Factors that were associated with significantly lower odds of good mental health were being raised by a foster carer and having lived in four or more homes since birth. Coffin [48] also trialled the SDQ with Western Australian Aboriginal youth but found issues with the length, number of words and level of English literacy, and concept understanding required to successfully complete the questionnaire. An adapted version of the SDQ was useful for augmenting observational improvements in the self-regulation, self-awareness, and socialisation skills of Indigenous youth after their participation in an equine assisted learning program. From New Zealand, Clark, Johnson [40] also evaluated a referral-based free counseling support service for youth with mild to moderate mental health problems and reported reduced risk of clinically significant mental health concerns measured by SDQ (*p* < 0.001).

The Children’s Global Assessment Scale (C-GAS) is designed to measure the lowest level of functioning for a child or youth. It produces a scale from 1–100 with scores above 70 indicating a normal functioning [40]. This tool uses a variety of questions to retrieve this score, using both positively and negatively loaded questions. It has been assessed as valid and reliable for general youth populations but has not been validated for Indigenous youth. From New Zealand, Clark, Johnson [40] evaluated a referral-based free counseling support service for youth with mild to moderate mental health problems and reported significant improvements in global social and psychiatric functioning measured by C-GAS.

#### Other Western tools which are reliable and or valid for Indigenous populations

The Patient Health Questionnaire 9 (PHQ-9 and adapted a-PHQ-9)^1^ are measures of depressive symptoms in patients and use a numerical score. The original tool was designed without consultation with Indigenous communities; however, the a-PHQ-9 has been deemed culturally acceptable with Indigenous Australian adults [58]. The questions and language are not strengths-based. Harriss [49] found that overall, the adapted PHQ-9 was straight forward and well-accepted by staff and youth as part of the Australian Yarrabah Young Person’s Health Check in north Queensland, but primary healthcare staff recommended more preparatory information and promotion and removal of the question about suicidal ideation as culturally inappropriate. Using the clinician-administered PHQ-9, Harriss, Kyle [49] found that one-in-five Yarrabah young people had moderate–severe symptoms or self-harm ideation in the previous 2 weeks; they were referred to the mental health service and followed up by trained staff.

The Kessler Distress Scale abridged version (K6 +) and other variants (K10, K5) use a scalar model to assess a person’s severity of distress. Participants are asked six questions that are not strengths-based regarding feelings of sadness, nervousness, hopelessness etc. The tool has been validated with Indigenous adults, but not with youth. However, Mitchell and Beals [42] found that with native American youth in two Northern Plains tribal reservations and a Southwestern tribal reservation, the Kessler-6 (K-6) was a useful complement to more traditional clinical decisions of presence or absence of disorders, and claim that during their study, there was no concern regarding the cultural validity of its use [42]. The K-6 results helped clinicians to understand the impact of psychological disorders more comprehensively, and to make more informed treatment recommendations and plans.

The Substance Abuse Choices Scale (SACS) was reported in two New Zealand studies. SACS is a scale that gives a score out of 20 regarding the consequences and use (and abuse) of substances and other addictive behaviours. In its design, it is focused on identifying the problems associated with substance use and addictive behaviours. From New Zealand, Clark, Johnson [40] evaluated a referral-based free counseling support service for youth with mild to moderate mental health problems and reported reduced use and impact of drugs or alcohol measured by SACS (*p* < 0.001). Goodyear-Smith, Corter [56] used SACS as a secondary screening tool, that was triggered when New Zealand youth (mainly female Maori youth) rated positive responses on the YouthCHAT tool regarding alcohol and drug use. It has been accepted for use within New Zealand Maori and Pacific communities [40, 56].

The Generalised Anxiety Disorder 7 (GAD-7) was used in New Zealand as a secondary screening tool. The GAD was triggered when mainly female Maori youth participants rated positive responses on the YouthCHAT tool regarding anxiety [56]. The GAD-7 tool is not strengths-based but was useful for identifying the three/30 youth who scored in the positive range for general anxiety disorder.

The Alcohol, Smoking and Substance Involvement Screening Test (ASSIST) for smoking was also used in New Zealand as a secondary screening tool, that was triggered when mainly female Maori youth participants rated positive responses on the YouthCHAT tool regarding smoking [56]. Also not strengths-based, the tool was nevertheless useful for identifying those who had risk of health and other problems from current use. The ASSIST was later replaced by the youth-specific SACS for alcohol and drug screening [56].

ED presentations and hospital admissions with a primary mental health diagnosis – were used as a measure of the extent of mental illness in child and youth clients of four Australian ACCHOs in New South Wales. Williamson, Skinner [51] found that over a median of 6-year follow-up, there were 96 ED presentations affecting 62 children (10.7/1000 person-years) and 49 hospitalisations for mental health conditions affecting 34 children (5.5/1000 person-years). Presentations and admissions increased with age. By linking population health datasets, they found that risk factors for ED presentation were living in foster care; high baseline child emotional/ behavioural problems; and caregiver chronic health conditions. Hospitalisations significantly increased when caregivers were unemployed and/or had chronic health problems.

## Discussion

Indigenous researchers have called for data to be conceptualised and framed through strengths-focussed values that recognise the capacities and capabilities of Indigenous peoples [59]. Of the 21 papers reviewed, it became clear that some of the tools are significantly more equipped and more appropriate for use within Indigenous youth communities and for measuring wellbeing. The results found only four of the 14 individual wellbeing instruments had been developed specifically for Indigenous populations and hence are likely to better reflect Indigenous relational concepts of wellbeing. However, only one of the Indigenous-developed tools encompassed solely strengths-based constructs (the ACHWM) and the other three included a combination of strengths- and deficit-focussed constructs. The Strong Souls tool was not considered to be reliable or valid with Indigenous Australians from other parts of the nation [62]. Nuanced understandings of Indigenous wellbeing across countries and places within countries also mean that the relevance of measurement instruments might need to be tested with Indigenous children and youth in different places. For example, Young et al. [e.g. 38, 46, 52] assured that the test–retest validity of the ACHWM meant that Aboriginal communities across Canada could use this measure with confidence, but advised that its relevance for Indigenous children in other regions of the world would need to be assessed [53]. The other 10 measurement instruments were standard Western tools, two of which had been adapted for Australian Indigenous populations [48, 49], and four tools had been translated into an Indigenous (Māori) language [55, 56].

Only four of the 14 wellbeing measures measured solely strength-based domains of wellbeing and four measured a mixture of strengths and problems. However, even for those tools that focussed on relational strengths-based concepts, none measured all five domains of the relational, multi-levelled construct of Indigenous wellbeing defined by McClintock, King [13]: emotions, motivations, spirituality, relationships and cultural connection. Only one instrument encompassed an aspect of cultural wellbeing such as language, cultural knowledge and cultural practice domains; Strong Souls included a measure of cultural resilience [43]. Only one measurement instrument encompassed an aspect of spiritual wellbeing such as spirituality, ancestors and land domains; ACHWM included a measure of spiritual health [38, 46, 47, 52, 53]. Finally, only four instruments encompassed aspects of collective or relational wellbeing with family, kinship, tribal, and community domains – the C-GAS (psychosocial functioning at home, at school, and with peers), the ORS (relational wellbeing, social wellbeing, and global wellbeing), SRS (the therapeutic relationship, global rating) and SDQ (peer problems, prosocial behaviour) [40, 44, 45, 54, 63].

In contrast, the other six measurement instruments focused on deficits, dubbed by Walter [35] as the five ‘Ds’ of data about Indigenous peoples: disparity, deprivation, disadvantage, dysfunction and difference. These measurement instruments were designed to measure pathologies [37] and tended to focus on individual characteristics – a significantly more Westernised approach to wellbeing [1]. Specifically, each of these scales comprise indicators of ill-being (e.g., depression, suicide ideation/risk, impulsivity and anxiety); therefore, they provide little meaningful insight into *well*being; a positive concept that implies more than the absence of pathology or illness. Use of these tools as reported in the included studies, however, had beneficial outcomes for Indigenous youth such as referrals to the mental health service and followed up by trained staff. This review is not intended to diminish the impact of these tools, but rather make the distinction between which tools are most effective at measuring *well*being of Indigenous youth, rather than pathological issues.

Fourteen of the 21 publications described child or youth self-reported outcomes, with one exhibiting both participant-reported and parent and clinician-reported outcomes [38, 42, 43, 46, 47, 54–57, 64]. Six publications described non-child and youth-reported outcomes, and were completed through parent, carer or clinician reporting or assessment on behalf of a child or youth [40, 44, 45, 49, 51, 63].

Our finding that nine of the included 21 publications reported the reliability and/or validity of measurement instruments for the target Indigenous youth population suggests that there have been some advances in determining the reliability and validity of instruments in the last 14 years [63]. At that time, Williamson et al. [9] reported that few Indigenous youth mental health studies used measurement instruments with previously determined reliability or validity. There was no consistency in the types of reliability or validity reported, rendering comparison impossible. Further Australian research studies to develop and validate new Indigenous child and youth wellbeing measures are in process [65–67].

Clearly the measurement of Indigenous youth wellbeing is complex and there are no universally appropriate measurement tools that are ideal for every Indigenous youth population group. The complexity makes it difficult for primary healthcare services to make decisions about how best they can measure the wellbeing of their Indigenous child and youth clients. These issues are becoming more prominent in view of the global disruption caused by the COVID-19 pandemic to children’s and youth education and wellbeing [68, 69]. Further research is needed to develop new Indigenous-specific tools or refine existing Indigenous-developed tools to ensure they are validated for different populations and purposes, and to adapt existing Western tools for cross-culturally application.

Limitations of this review include location of all authors in Australia, so it is possible that relevant papers and reports from Canada, New Zealand, and the United States were missed. However, the review was based on a systematic review of the published and grey literature using pre-defined terms and conducted by an experienced librarian. We excluded studies that used measurement tools with Indigenous children and youth in non-primary healthcare settings such as through digital apps e.g. [70], in schools e.g. [71, 72], child protection e.g. [73] and juvenile justice settings e.g. [74], and focussed instead solely on those used in primary healthcare settings. Primary healthcare settings are the most accessible form of care in many Indigenous communities and as described by the World Health Organisation, is the most appropriate space to measure wellbeing of youth. We also excluded studies that focussed on organisational performance criteria for the youth wellbeing services of primary healthcare services, e.g. [76], on the basis that they did not measure the wellbeing of Indigenous children or youth.

Finally we acknowledge that there are critiques of some of our desirability criteria. For example, measurement instruments developed specifically by or for Indigenous populations have been critiqued as being unable to be norm-referenced to the general population and hence potentially perpetuating unhelpful assumptions of difference and perceptions of disparity (e.g. [37]). However, in primary healthcare settings, benchmarking with the general population was not the priority need. Similarly, administration by self-report is often considered problematic because it is commonly associated with bias; however, there are effective methods for controlling such bias [22]. The international evidence suggests that each of these criteria is worthy of consideration in the assessment of the value of a measurement instrument.

## Conclusions

The review is led by an Indigenous researcher and contributes to identifying measurement instruments that are developed for or have been applied in primary healthcare services to measure the wellbeing of Indigenous children and youth. It was conducted at a point in time, and has become particularly pertinent in response to the concerns by Indigenous Australian primary healthcare services about the effects of COVID-19 on youth wellbeing, and the call by the International Group on Indigenous Health Measurement for a robust literature review of relevant wellbeing factors across Indigenous participant samples [13]. The review found that there is a diversity of tools available for use, but only a few that can give a holistic and culturally appropriate measure of wellbeing. Those that best fitted our desirability criteria were the ACHWM, YouthCHAT, ORS, SRS, SDQ, GAD and WASC-Y. Given the importance of Indigenous youth wellbeing, more needs to be done to adapt existing Western tools cross-culturally for use with indigenous youth or further research and refine Indigenous-developed tools to ensure they are validated for different Indigenous youth population groups, needs and settings. This review supports several Australian research studies that are currently underway to develop measures/ screening tools to assess the wellbeing of Indigenous children and youth [65–67].

## Supplementary Information

**Additional file 1.**

## Data Availability

All data generated or analysed during this study are included in this published article [and its supplementary information files].

## Abbreviations

ACCHO: Aboriginal Community Controlled Health Organisation
ACHWM: Aboriginal Children’s Health and Well-Being Measure
ASSIST: Alcohol, Smoking and Substance Involvement Screening Test
CANZUS: Canada, Australia, New Zealand and the United States
C-GAS: Children’s Global Assessment Scale
e-CHAT: Electronic Case-finding and Help Assessment Tool
ED: Emergency Department
GAD-7: Generalised Anxiety Disorder 7
K-6: Kessler Distress Scale
NAHS: National Aboriginal Health Strategy Working Party
non-PRO: Non-patient-reported outcomes
ORS: Outcome Rating Scale
PHQ-9: Patient Health Questionnaire 9
PROs: Patient-reported-outcomes
SACS: Substance Abuse Choices Scale
SDQ: Strengths and Difficulties Questionnaire
SEWB: Social and emotional wellbeing
SRS: Session Rating Scale
WASC-Y: Westerman Aboriginal Symptoms Checklist Youth
YouthCHAT: Youth Case-finding and Help Assessment Tool
