# Supplementary material for: Counting what counts: a systematic scoping review of instruments used in primary healthcare services to measure the wellbeing of Indigenous children and youth
Source: BMC Prim Care. 2023 Feb 17;24:51. doi: 10.1186/s12875-023-02001-z (PMC9936129; doi:10.1186/s12875-023-02001-z)
Supplement: Supplementary file 1 — Additional file 1. [file 12875_2023_2001_MOESM1_ESM.zip › Supplementary table 1.docx]

**Supplementary document 1: Measurement instruments**

| **Measure / Scale** | | **SEWB & Mental Health Domains Assessed** | **Application / Study Reviewed** | **Sample** |  | **Type** | **PRO/non-PRO**  **(patient reported outcome)** | **Reliability** | **Validity** |
| --- | --- | --- | --- | --- | --- | --- | --- | --- | --- |
| 1 | Aboriginal Children’s Health and Well-Being Measure (ACHWM) | Holistic health including spiritual, emotional, physical and mental health (Young et al., 2013) | Reported on development of the measure (Young et al., 2013); | 38 First Nations Canadian children and youth, 8-17 years |  | Indigenous Developed | PRO | Not reported for this study | Content Validity |
|  |  |  | Assessed convergent validity of the measure (N. L. Young et al., 2015) | 48 First Nations Canadian children and youth, 7-19 years |  | Indigenous Developed | PRO | Not reported for this study | Convergent validity (N. L. Young et al., 2015) |
|  |  |  | Assessed consistency and accuracy of children and youth’s interpretations of the ACHWM and established face validity (Nancy L. Young et al., 2015) | 9 First Nations Canadian children and youth, 8-18 years, and 9 caregivers |  | Indigenous Developed | PRO | Not reported for this study | Face validity |
|  |  |  | Evaluated a screening process embedded in the ACHWM (N. L. Young et al., 2016) | 293 First Nations Canadian children and youth, 8-18 years |  | Indigenous Developed | PRO | Not reported for this study | Not reported for this study |
|  |  |  | Evaluated reliability of the ACHWM (Nancy L. Young et al., 2016) | 256 First Nations Canadian children and youth, 8-18 years |  | Indigenous Developed | PRO | Cronbach's α = 0.93  Test-retest = ICC of 0.94 | Not reported for this study |
|  |  |  | Use of ACHWM to evaluate outdoor adventure leadership experience (Usaba, 2019) | 30 Indigenous youth |  | Indigenous developed | PRO | As above | As above |
| 2. | Alcohol, Smoking and Substance Involvement Screening Test (ASSIST) for smoking | Frequency of substance use and associated problems | Electronic version of Youthchat allows branching logic – positive responses to smoking, alcohol and drugs lead participants to complete ASSIST (F. Goodyear-Smith, Corter, & Suh, 2016; Felicity Goodyear-Smith et al., 2017) | 30 New Zealand youth <25 years, including 27 Māori participants |  | Standard but translated into Māori language (Te Reo) | PRO | Not reported for this study | Not reported for this study |
| 3. | Children’s Global Assessment Scale (C-GAS) | Psychosocial functioning (at home, at school, and with peers) | Used to measure provider assessment of psychosocial functioning among participating children and adolescents (Clark et al., 2014) | 581 culturally diverse New Zealand youth aged 10–24, including 182 Māori youth |  | Standard | Non-PRO  Provider assessed | C-GAS is a reliable measure | C-GAS is a valid measure utilized in New Zealand Maori and Pacific, but has not been validated for this population. |
| 4. | ED presentations and hospital admissions with a primary mental health diagnosis (Williamson et al., 2018) | Hospitalisable mental health diagnosis | Reported mental health-related emergency department (ED) presentations and hospitalisations, and associated child and family characteristics, in children recruited through four Aboriginal Community Controlled Health Organisations | 1476 Aboriginal Australian children aged 0–17 years |  | Standard | Non-PRO | Not reported for this study | ICD codes used to identify mental health-related presentations and admissions with a mental health-related principal diagnosis code for initial episode of care. |
| 5. | Generalised Anxiety Disorder 7 (GAD-7) | Generalised anxiety | Positive responses to anxiety on Youthchat led to extra assessment using GAD-7 among youth in a screening and intervention for lifestyle risk factors and mental health issues (F. Goodyear-Smith et al., 2016; Felicity Goodyear-Smith et al., 2017) | 30 New Zealand youth under the age of 25, including 27 Māori participants |  | Standard but translated into Māori language (Te Reo) | PRO | Not reported for this study, but other studies of reliability | Not reported for this study |
| 6. | Kessler Distress Scale abridged version (K6+) | Mental health problems and severity, including feeling sad, nervous, restless or fidgety, hopeless, everything is an effort, or worthless | Pilot tested to assess its reliability and validity with Indigenous Australian youth (Thomas, Cairney, Gunthorpe, Paradies, & Sayers, 2010) | 67 Indigenous Australian students aged 16 – 20.5 |  | Standard | Non-PRO | Cronbach's α = 0.7 | Not reported for this study |
|  |  |  | Examined psychometric properties and utility in predicting mood disorders among Native American youth (Mitchell & Beals, 2011) | 3,084 Native American youth ages 15–54 years |  | Standard | Non-PRO | Cronbach's α = .83 | Content validity Incremental validity |
| 7. | Outcome Rating Scale (ORS) | ORS assesses personal wellbeing; relational wellbeing; social wellbeing; and global wellbeing. | Reported YouthLink framework empirical research design where 40 Aboriginal Australian clients completed client feedback monitoring measures between 2014 and 2016 (Sabbioni et al., 2018). | Young people aged 13 to 24 years of age. |  | Standard | PRO | Reliability reported in other studies. Principles are culturally appropriate and aligned with Aboriginal perspectives | Not cross-culturally validated but strong face validity and clinical utility. Criterion validity against the HoNOS. |
| 8. | Patient Health Questionnaire 9 (PHQ-9) | Depressive symptoms | Positive responses to depression on Youthchat led to extra PHQ-9 assessment in a screening and intervention for lifestyle risk factors and mental health issues (F. Goodyear-Smith et al., 2016; Felicity Goodyear-Smith et al., 2017) | 30 New Zealand youth under the age of 25, including 27 Māori participants (F. Goodyear-Smith et al., 2016) |  | Standard (translated into a Māori language (Te Reo) version) | PRO | Not reported for this study | Not reported for this study |
|  |  |  | The aPHQ-9 measures depressive symptoms as a score, ranging from 0 (absence of depressive symptoms), 1–4 (minimal), 5–9 (mild), 10–14 (moderate), 15–19 (moderately severe) and 20–27 (severe) (Harriss et al., 2018) | 122 youth aged 15-25 years from Yarrabah Aboriginal community |  | Adapted specifically for use in Aboriginal communities of Central Australia and was found to be culturally acceptable there. | Non-PRO | Not reported in this paper, but other studies of reliability | aPHQ-9 screening was straight forward and well accepted by staff and youth. The aPHQ-9 has now been validated, and found to be culturally appropriate and acceptable for Indigenous adults in 10 diverse Indigenous communities across Australia (Hackett et al., 2019) |
| 9. | Session Rating Scale (SRS) | Therapeutic alliance - 4 questions on different element of treatment session (relationship, goals, topics, global rating). | Reported YouthLink framework empirical research design where 40 Aboriginal clients completed client feedback monitoring measures between 2014 and 2016 (Sabbioni et al., 2018). | Young people aged 13 to 24 years of age. |  | Standard organisational measure | PRO | Not reported for this study, but reliability established in other studies | Not cross-culturally validated but strong face validity and clinical utility |
| 10. | Strengths and Difficulties (SDQ) | Strengths and difficulties including 5 scales for conduct problems, hyperactivity- inattention, emotional symptoms, peer problems, and prosocial behaviour | SDQ used to measure psychosocial functioning among participating children and adolescents (Clark et al., 2014) | *n=*581 culturally diverse New Zealand youth aged 10–24, including 182 Māori youth |  | Standard | PRO | Reliability has been well established | Validity has been well established -SDQ has been identified as a preferred rating scale among New Zealand young people |
|  |  |  | Assesses the acceptability and face validity of SDQ among Aboriginal parents and workers (Williamson et al., 2010) | Aboriginal Australian parents, *(n*=15) and Aboriginal staff/workers (*n*=32) |  | Standard | PRO and non-PRO (carer reported version) | SDQ has previously been found to be acceptable, reliable and valid among the SEARCH cohort | Face/content validity |
|  |  |  | Examines the construct validity of the standard carer reported SDQ for Aboriginal children in Australia (Williamson et al., 2014) | *n*=717 urban Australian Aboriginal Australian children and adolescents aged 4-17 |  | Standard | Non-PRO (Carer reported) | Cronbach's α  = .85 | Convergent Validity  Construct Validity (Confirmatory Factor Analysis) |
|  |  |  | Identifies factors associated with ‘good’ mental health among Aboriginal children living in urban communities in New South Wales, Australia (Williamson et al., 2016). | **N =** 1005 Aboriginal children aged 4–17 years |  | Standard | Non-PRO (Carer reported) | SDQ has previously been found to be acceptable, reliable and valid among the SEARCH cohort | Not reported for this study |
|  |  |  | To evaluate alternative therapy for Aboriginal Australian youth in the areas of grief, loss, and trauma, through an equine assisted learning program (Coffin, 2019) | 270 participants aged 6–25 years old engaged in a minimum of 6-weeks of equine assisted learning |  | Adapted | Non-PRO | Not reported for this study | Issues with the length, number of words and level of English literacy, and concept understanding required to successfully complete the questionnaire |
| 11. | Strong Souls | Social and emotional wellbeing, including problems related to depression, anxiety, suicide risk and levels of resilience | Reports on the development and validation of the Strong Souls scale for Indigenous Australian adolescents (Thomas et al., 2010) | Australian Aboriginal youth in NT aged 16 – 20.5 years, *n=*67 in pilot study, and *n=*361 in the main study |  | Developed for Indigenous youth | PRO | Cronbach's α  = .70 | Face Validity  Content Validity Discriminant Validity  Convergent Validity  Exploratory Factor Analysis |
|  |  |  | Assesses the psychometric properties of Strong Souls (Gorman, 2021) | Australian Aboriginal youth in NSW |  | Developed for Indigenous youth | PRO | Not considered reliable for Aboriginal youth | N/A |
| 12. | Substance Abuse Choices Scale (SACS) | Substance use, frequency of use,  addictive behaviours, harms and consequences of substance use | SACS used to measure substance use among participating children and adolescents (Clark et al., 2014) | *n=*581 culturally diverse New Zealand youth aged 10–24, including 182 Māori youth |  | Standard | PRO | SACS is a reliable scale developed in New Zealand | Appears to be an acceptable screening tool for Maori and Pacific communities |
|  |  |  | Used to assess for substance use among youth in a screening and intervention for lifestyle risk factors and mental health issues (F. Goodyear-Smith et al., 2016) | 30 New Zealand youth under the age of 25, including 27 Māori participants |  | Standard (translated into a Māori language (Te Reo) version) | PRO | Not reported for this study | Not reported for this study |
| 13. | Westerman Aboriginal Symptoms Checklist- Youth (WASC-Y) | Depression, suicidal behaviours, drug and alcohol use, impulsivity, anxiety and cultural resilience. | Pilot tested to assess its reliability and validity with Indigenous Australian youth (Thomas et al., 2010). | Australian Aboriginal youth ages 16 – 20.5 years, *n=*67 |  | Indigenous Developed | PRO | Cronbach's α  = .70 (not all the reliabilities for WASC-Y subscales could be established) | Face Validity  Discriminant Validity |
| 14. | YouthCHAT | Smoking, drinking or other drugs; gambling; depression; anxiety; sexual orientation; sexually active; risky sexual behaviour: STI; pregnancy; unwanted sex; exposure to abuse; anger control; physical inactivity. | Community-based participatory research approach. Quantitative and qualitative – analysis of screening results and survey (F. Goodyear-Smith et al., 2016; Felicity Goodyear-Smith et al., 2017). | 30 youth < 25 years completed YouthCHAT. Twenty-eight (93 %) were female, and 27 (90 %) were Māori, with the remainder NZ European. |  | Developed for Māori and disadvantaged youth | PRO | High acceptability (M =8.29/10) | Easy to use, helped them think about and identify problems, talk with their doctor, and assisted their doctor to be aware of issues. |
|  |  |  | Comparison of YouthCHAT with a clinician interview assessment (Thabrew, 2019) |  |  |  |  |  |  |

Clark, T. C., Johnson, E. A., Kekus, M., Newman, J., Patel, P. S., Fleming, T., & Robinson, E. (2014). Facilitating access to effective and appropriate care for youth with mild to moderate mental health concerns in new zealand. *Journal of Child & Adolescent Psychiatric Nursing, 27*(4), 190-200. doi:<https://dx.doi.org/10.1111/jcap.12095>

Coffin, J. (2019). The Nguudu Barndimanmanha Project-Improving Social and Emotional Wellbeing in Aboriginal Youth Through Equine Assisted Learning. *Front Public Health, 7*, 278. doi:10.3389/fpubh.2019.00278

Goodyear-Smith, F., Corter, A., & Suh, H. (2016). Electronic screening for lifestyle issues and mental health in youth: a community-based participatory research approach. *BMC Medical Informatics & Decision Making, 16*(1), 140. doi:<https://dx.doi.org/10.1186/s12911-016-0379-z>

Goodyear-Smith, F., Martel, R., Darragh, M., Warren, J., Thabrew, H., & Clark, T. C. (2017). Screening for risky behaviour and mental health in young people: the YouthCHAT programme. *Public Health Reviews, 38*(1), 20. doi:10.1186/s40985-017-0068-1

Hackett, M., Teixeira-Pinto, A., Farnbach, S., Glozier, N., Skinner, T., & Askew, D. (2019). Getting it Right: validating a culturally specific screening tool for depression (aPHQ-9) in Aboriginal and Torres Strait Islander Australians. *Medical Journal of Australia, 211*, 24-30.

Harriss, L. R., Kyle, M., Connolly, K., Murgha, E., Bulmer, M., Miller, D., . . . McDonald, M. (2018). Screening for depression in young Indigenous people: Building on a unique community initiative. *Australian Journal of Primary Health, 24(4)*, 343-349. doi:<http://dx.doi.org/10.1071/PY18006>

Mitchell, C. M., & Beals, J. (2011). The utility of the Kessler Screening Scale for Psychological Distress (K6) in two American Indian communities. *Psychological Assessment, 23*(3), 752-761. doi:<https://dx.doi.org/10.1037/a0023288>

Sabbioni, D., Feehan, S., Nicholls, C., Soong, W., Rigoli, D., Follett, D., . . . Waters, F. (2018). Providing culturally informed mental health services to aboriginal youth: The youthlink model in western australia. *Early Intervention in Psychiatry, 12*(5), 987-994. doi:<http://dx.doi.org/10.1111/eip.12563>

Thomas, A., Cairney, S., Gunthorpe, W., Paradies, Y., & Sayers, S. (2010). Strong Souls: Development and validation of a culturally appropriate tool for assessment of social and emotional well-being in Indigenous youth. *Australian and New Zealand Journal of Psychiatry, 44*(1), 40-48. doi:10.3109/00048670903393589

Williamson, A., D'Este, C., Clapham, K., Redman, S., Manton, T., Eades, S., . . . Raphael, B. (2016). What are the factors associated with good mental health among Aboriginal children in urban New South Wales, Australia? Phase I findings from the Study of Environment on Aboriginal Resilience and Child Health (SEARCH). *BMJ Open, 6*(7), e011182. doi:10.1136/bmjopen-2016-011182

Williamson, A., McElduff, P., Dadds, M., D' Este, C., Redman, S., Raphael, B., . . . Eades, S. (2014). The construct validity of the strengths and difficulties questionnaire for Aboriginal children living in urban New South Wales, Australia. *Australian Psychologist, 49*(3), 163-170. doi:10.1111/ap.12045

Williamson, A., Redman, S., Dadds, M., Daniels, J., D'Este, C., Raphael, B., . . . Skinner, T. (2010). Acceptability of an emotional and behavioural screening tool for children in Aboriginal Community Controlled Health Services in urban NSW. *Australian and New Zealand Journal of Psychiatry, 44*(10), 894-900. doi:<http://dx.doi.org/10.3109/00048674.2010.489505>

Williamson, A., Skinner, A., Falster, K., Clapham, K., Eades, S. J., & Banks, E. (2018). Mental health-related emergency department presentations and hospital admissions in a cohort of urban Aboriginal children and adolescents in New South Wales, Australia: findings from SEARCH. *BMJ Open, 8*(11), e023544. doi:10.1136/bmjopen-2018-023544

Young, N. L., Jacko, D., Wabano, M. J., Hawthorne, L., Seabrook, S., Wabanosse, S., & Usuba, K. (2016). A screening mechanism to recognize and support At-Risk aboriginal children. *Canadian Journal of Public Health, 107*(4-5), e399-e403. doi:10.17269/CJPH.107.5539

Young, N. L., Wabano, M. J., Burke, T. A., Ritchie, S. D., Mishibinijima, D., & Corbiere, R. G. (2013). Process for creating the Aboriginal Children's Health and Well-being Measure (ACHWM). *Canadian Journal of Public Health, 104*(2), e136-e141. Retrieved from <https://www.scopus.com/inward/record.uri?eid=2-s2.0-84876552287&partnerID=40&md5=f71b9e45874927fb3716489a6b877d3a>

Young, N. L., Wabano, M. J., Ritchie, S. D., Burke, T. A., Pangowish, B., & Corbiere, R. G. (2015). Assessing children's interpretations of the Aboriginal Children's Health and Well-Being Measure (ACHWM). *Health and quality of life outcomes, 13*(1), 105. doi:10.1186/s12955-015-0296-3

Young, N. L., Wabano, M. J., Usuba, K., Mishibinijima, D., Jacko, D., & Burke, T. A. (2016). Reliability of the Aboriginal Children's Health and Well-Being Measure (ACHWM). *Springer Plus, 5*(1), 1. doi:10.1186/s40064-016-3776-y

Young, N. L., Wabano, M. J., Usuba, K., Pangowish, B., Trottier, M., Jacko, D., . . . Corbiere, R. G. (2015). Validity of the Aboriginal children's health and well-being measure: Aaniish Naa Gegii? *Health & Quality of Life Outcomes, 13*, 148. doi:<https://dx.doi.org/10.1186/s12955-015-0351-0>
