# Supplementary material for: Counting what counts: a systematic scoping review of instruments used in primary healthcare services to measure the wellbeing of Indigenous children and youth
Source: BMC Prim Care. 2023 Feb 17;24:51. doi: 10.1186/s12875-023-02001-z (PMC9936129; doi:10.1186/s12875-023-02001-z)
Supplement: Supplementary file 1 — Additional file 1. [file 12875_2023_2001_MOESM1_ESM.zip › Supplementary table 2 - Data Extraction Table.docx]

| **Table 2: Included publications that described the development or use of wellbeing measurement instruments with indigenous children and youth** | | | | | | |
| --- | --- | --- | --- | --- | --- | --- |
| **1^st^ Author/ Publication Year and Type** | **Study aims** | **Country**  **Target group** | **Sample Size**  **Study setting** | **Study design** | **Outcome measures**  **Screening/measurement tools & reported reliability/validity with study population** | **Outcomes** |
| **Clark (2014) [27]**  **Journal article** | **Study aims**: To evaluate a referral-based facilitated access to free counseling support for youth with mild to moderate mental health problems. | **Country:** New Zealand  **Target group**: Youth with mild–moderate mental health concerns, including Māori youth. | **Sample Size:** 581 culturally diverse youth aged 10–24, including Māori (n=182, 31.4%).  **Study setting:** Referrals were received from any primary care organization, schools, and community organizations; secondary services, including self-referrals from family/whanau (extended family); and young people within the region. The counseling providers utilised a range of therapy modalities in a range of settings, including schools, homes, and community-based services that were convenient to the young person. | **Study design:** A quasi-experimental pre-/post intervention design. | **Outcome measures:** Clinical outcomes related to wellbeing and substance use were measured pre- and post-intervention.  **Screening/measurement tools:**  Strengths and Difficulties Questionnaire (SDQ);  Substance Abuse Choices Scale (SACS);  Children’s Global Assessment Scale (C-GAS) [provider assessed]  **Reported reliability/validity with study population**: Baseline measures of well-being and substance use as measured by the Strengths and Difficulties Questionnaire (SDQ) and Substance Abuse Choices Scale (SACS) were completed on enrollment. Providers were asked to complete the Children’s Global Assessment Scale (C-GAS), which produces a Global Assessment of Functioning (GAF) score. All measures were repeated at the completion of the intervention at the final meeting with the contracted provider. | **Outcomes:** Those who completed reported significant improvements in global social and psychiatric functioning measured by C-GAS (*p* < .001); reduced risk of clinically significant mental health concerns measured by SDQ (*p* < .001); and reductions in the use and impact of drugs/alcohol measured by SACS (*p* < .001). Participants and their families/whanau reported that the interventions were safe and appropriate, with perceived increased skill development around coping and communication. |
| **Coffin (2019) [36]**  **Journal article** | **Study aims:** Alternative therapy for Aboriginal youth in the areas of grief, loss, and trauma, through an equine assisted learning program that focussed on self-concept, self-regulation, self-awareness, anxiety and depression, and sense of connectedness. | **Country developed:** Australia  **Target grou**p: Aboriginal youth | **Sample size:** Participants (*N* = 270) aged 6–25 years old engaged in a minimum of 6-weeks of equine assisted learning.  **Study setting:** The Equine Assisted Learning (EAL) model provides a vehicle for Aboriginal practitioners to provide culturally secure and appropriate interventions to improve mental, emotional, social, and spiritual wellbeing (14). The physical environment for therapy can help alleviate mistrust and stress and is commonly reported as a benefit in itself, aiding in relaxation and enabling a multi-sensory approach to therapy | **Study design:** Participants (*N* = 270) aged 6–25 years old engaged in a minimum of 6-weeks of equine assisted learning. Each session was 45–50 min duration and occurred on a weekly basis. Sessions were undertaken individually, in pairs and in groups, depending on the needs of the participant and the focus of the session goals. Qualitative examination of the participants included photography to capture the lived experiences of the participants throughout the program. In addition, a cultural and age appropriate adaptation of the Strength and Difficulties Questionnaire was trialed to track changes quantitively. | **Outcome measures:** In addition to the qualitative data collection CI Coffin developed and trialed a culturally secure paper based tool to assess changes in social and emotional wellbeing. The tool was based on the Strengths and Difficulties Questionnaire (SDQ) (32). The new tool was created for three distinct age groupings in a similar fashion to the SDQ (6–10, 11–16, and 17 years and over), however the questionnaire was only administered to the first two age groups due to low numbers in the third group  **Screening/measurement tools:** Modified Strengths and Difficulties Questionnaire (SDQ)  **Reported reliability/validity with study population:** The SDQ was trialed with a sample of participants however there were issues with the length, number of words and level of English literacy, and concept understanding required to successfully complete the questionnaire. | **Outcomes:** observed improvements in self-regulation, self-awareness, and socialization skills, evident from the photography recording and the questionnaire data. In addition parent and/or caregiver and teacher reported changes in behavior, self-regulation, and socialization skills were recorded. |
| **Goodyear-Smith (2016) [25]**  **Journal article** | **Study aims**: To tailor and pilot YouthCHAT, a youth programme for electronic screening and intervention for lifestyle risk factors and mental health issues, for use in a clinic catering for disadvantaged youth, assess its acceptability and utility, and develop a framework to scale-up its implementation. | **Country:** New Zealand  **Target group**: Youth attending a clinic specifically targeted for disadvantaged youth. | **Sample Size:** Thirty youth, under age 25 years completed YouthCHAT and the survey. Twenty-eight (93 %) were female, and 27 (90 %) were Māori, with the remainder NZ European.  **Study setting:** YouthCHAT was implemented in a health clinic co- located at a low-decile school with a high Māori population in rural New Zealand. Fifty percent who attend the clinic are non-school enrolled Māori youth, including teenage parents and unemployed. | **Study design:** Community-based participatory research approach.  Quantitative and qualitative – analysis of screening results and survey. | **Outcome measures: Youthchat -** lifestyle risk factors and mental health issues, for use in a clinic catering for disadvantaged youth. Four additional screening tools were activated when a positive response was triggered by the patient in YouthCHAT  **Screening/measurement tools:**  YouthCHAT - with  Alcohol, Smoking and Substance Involvement Screening Test (ASSIST) for smoking;  Substances and Choices Scale (SACS);  Patient Health Questionnaire for Depression (PHQ-9);  Generalised Anxiety Disorder screen (GAD-7).  **Reported reliability/validity with study population**: YouthCHAT screening results across 13 domains (smoking, drinking or other drugs; gambling; depression; anxiety; sexual orientation; sexually active; risky sexual behaviour: STI; risky sexual behaviour: pregnancy; unwanted sex; exposure to abuse; anger control; physical inactivity). All recruited youth were invited to complete a survey after their consultation. Measures included demographics (age, gender, ethnicity, employment status), number of YouthCHAT issues for which youth wanted help, Likert scores for acceptability and utility, and free text comments. | **Outcomes:** 27 (90 %) screened positive for at least one domain. Nineteen (67 %) had one to three issues. Sixteen (53 %) wanted help with at least one issue, either immediately or later. Patients gave YouthCHAT high acceptability ratings (M = 8.29/10), indicating it was easy to use, helped them think about and identify problems, talk with their doctor, and assisted their doctor to be aware of these issues. They liked that YouthCHAT kept them busy in the waiting room and gave them time to reflect on their responses, and what to discuss with their clinician. Clinicians felt that YouthCHAT was acceptable to young patients because it was electronic and reinforced their privacy. They indicated YouthCHAT identified problems that would not be identified in a normal consult, and improved consultations by making them faster. |
| **Goodyear-Smith 2017[1]** | **Study aims**: To describe YouthCHAT, electronic screening and intervention for lifestyle risk factors and mental health issues. | **Country:** New Zealand  **Target group**: Youth attending a clinic specifically targeted for disadvantaged youth, with particular focus on Māori youth. | **Sample Size:** Youth aged <25  **Study setting: a** health clinic co- located at a low-decile school with a high Māori population in rural New Zealand. | **Study design:** Description of Youthchat implementation. | **Outcome measures: Youthchat -** lifestyle risk factors and mental health issues. Four additional screening tools activated when a positive response was triggered by the patient in YouthCHAT  **Screening/measurement tools:**  YouthCHAT - with  Alcohol, Smoking and Substance Involvement Screening Test (ASSIST) for smoking;  Substances and Choices Scale (SACS);  Patient Health Questionnaire for Depression (PHQ-9);  Generalised Anxiety Disorder screen (GAD-7).  **Reported reliability/validity with study population**: | **Outcomes:** Patients indicated it was easy to use, helped them think about and identify problems, talk with their doctor, and assisted their doctor to be aware of these issues. |
| **Harriss et al. 2018 [32]**  **Journal article** | **Study aims:** This study describes the trialling of an adapted PHQ=9 measure of depression as part of the Yarrabah Young Person’s Health Check (YPC) in 2016. It reports the prevalence of depressive symptoms, examines local issues related to the use of the screening tool and proposes recommendations for future health screening. | **Country:** Australia  Target group: Yarrabah community residents aged 15–25 years | **Sample size:** 122 Yarrabah youth (35% of total youth who participated in YPC)  **Study setting:** Experienced health professionals conducted the aPHQ-9 assessment in a private area of the Gurriny Yealamucka Health Service clinic. Gurriny is an Aboriginal Community-Controlled Health Service. | **Study design:** Statistical analysis of screening results | **Outcome measures:** Depressive symptoms  **Screening measurement tool:** Adapted PHQ-9 depression screening tool. The aPHQ-9 measures depressive  symptoms as a score, ranging from 0 (absence of depressive  symptoms), 1–4 (minimal), 5–9 (mild), 10–14 (moderate), 15–19 (moderately severe) and 20–27 (severe). People were referred to  an onsite medical practitioner and the Social and Emotional  Wellbeing Team if they had aPHQ-9 scores _10, or if there was an indication of self-harm ideation in the previous 2 weeks. | **Outcomes:** One-in-five young people were found to have moderate–severe symptoms or self-harm ideation in the previous 2 weeks; they were referred to the mental health service. The aPHQ-9 screening process was found to be straightforward and well accepted by staff and youth. Importantly, it provided valuable ‘space’ to facilitate communication on sensitive issues and was a conduit for speedy referral and follow up by trained staff. |
| **Mitchell (2011) [30]**  **Journal article** | **Study aims**: This study used data from a large epidemiological study of two tribal groups of American Indians living on or near their reservations to examine two research questions: 1. Is the K6 psychometrically appropriate for use in this population? 2. How might the K6 add information for researchers and clinicians about the severity of a diagnosis that moves beyond the presence/absence of emotional, substance use and physical disorders? | **Country developed:** United States  **Target group**: Two American Indian populations 15-54 years old, living on or near their home reservations (Northern Plains tribes and a Southwestern tribe living on or within 20 miles of their reservations at the time of sampling) | **Sample Size:** n= 3,084  **Study setting:** Tribal rolls formed the sampling universe; these records list all individuals meeting minimal requirements for recognition as tribal members. tribal enrolment coincided with eligibility for IHS services—the major health care provider in the rural communities involved and a major focus of the services research. | **Study design:**  Stratified random sampling procedure used in community-based study  Interview that included a version of the Composite International Diagnostic Interview (CIDI) and the K6 | **Outcome measures:** The appropriateness of K6 as a screening measure in tribal populations and the incremental validity and potential clinical utility of the K6  **Screening/measurement tools:** K6 and CIDI  **Reported reliability/validity with study population**: K6 was shown to be an appropriate screening and severity measure of mood disorders in the two samples | **Outcomes:**  The inclusion of scalar assessment such as K6 can be an important a complement to more traditional dichotomous decisions of presence or absence of disorder in more thoroughly understanding the impact of psychological disorders and making more informed treatment recommendations and plans |
| Sabbioni, et al. (2018) [31] | Study aims: This study describes and documents the effectiveness of the culturally sensitive model within YouthLink, a state-wide mental health service program in Western Australia for young people. | **Country developed:** Australia  **Target group:** providing culturally informed mental health services to Aboriginal youth | Sample Size: young people aged 13 to 24 years of age.  **Study setting:** Western Australia/ Aboriginal communities | Study design: A mixed-method design including a descriptive approach reporting on the YouthLink framework and an empirical research design where 40 Aboriginal clients completed client feed- back monitoring measures between 2014 and 2016. | **Outcome measures**: Outcome Rating Scale and Session Rating Scales (ORS and SRS) feedback measure for improving client outcomes.  **Screening/measurement tools:** The ORS assesses well-being with 4 questions about personal well-being, relational well-being, social well-being, global wellbeing. The SRS asks about therapeutic alliance with 4 questions on elements of the treatment session (relationship, goals, topics, global rating). Instructions ask the young person to place a mark on 10-cm line. The score is a summation of the marks to the nearest millimetre.  **Reported reliability/validity with study population:** They have not yet been cross-cultural validated, but the principles of feedback monitoring can be considered more culturally appropriate and more aligned with Aboriginal perspectives than standardised measurement approaches. ORS data are entirely in line with normative data collated in non-Indigenous clients suggesting that the measure does not discriminate against Aboriginal people. | **Outcomes:** this study was not designed to measure impact although there is evidence showing that such measures can increase treatment efficacy  Results of the ORS were also compared against scores on the Health of the Nation Outcome Scales (HoNOS). This clinician-rated scale is a standardised tool which assesses mental health with 12 items on a 5-point scale (ranging from 0 to 4).  The ORS and SRS represent a valuable alternative approach to standardized measures, and show strong face validity and clinical utility. The measures are simple, brief and appropriate, and are successful in capturing clients’ perspectives about their treatment progress and therapeutic bond. |
| **Thomas (2010) [29]**  **Journal article** | **Study aims**: The aim of the study was to develop and validate an appropriate tool to assess the social and emotional well-being of Indigenous adolescents participating in the longitudinal Aboriginal Birth Cohort (ABC) Study. | **Country developed:** Australia  **Target group**: Indigenous populations representing significant cultural and linguistic diversity from a range of urban, rural and remote communities across northern Australia | **Sample Size:** Pilot study: 67 students, aged between 13 and 19 years  Main study: 361 participants, aged between 16 and 20.5 years (n = 169, 47% male and n=192, 53% female)  **Study setting:** Pilot study: students from a local boarding high school in Darwin | **Study design:** Pilot study: supervised study session at school  Main study: participants completed an extended battery of health measures that included the Strong Souls questionnaire | **Outcome measures:** Construct validity of Strong Souls  **Screening/measurement tools:** WASC-Y Strong Souls  **Reported reliability/validity with study population**: Strong Souls demonstrated sound construct validity and reliability and appropriateness as a tool for screening SEWB among Indigenous young people in the Northern Territory. | **Outcomes:** Factor analysis produced a 25-item, four-factor model accounting for 34.5% of the variance. |
| **Williamson (2010) [33]**  **Journal article** | **Study aims**: To assess the acceptability and face validity of the Strengths and Difficulties Questionnaire (SDQ) for Australian Indigenous children. | **Country:** Australia  **Target group**: Aboriginal parents and Aboriginal staff who may, have administered or may be asked to administer the SDQ. | **Sample Size:** A total of 47 people participated in the study, including: Aboriginal parents, (n=15); Aboriginal research assistants and youth workers, (n=5); and, Aboriginal Medical Service (AMS) staff and other local Aboriginal workers such as Aboriginal education officers, (n=27). It was not possible to recruit adolescents.  **Study setting:** Three Aboriginal community controlled health services (ACCHOs) located in the greater Sydney region: Aboriginal Medical Service Western Sydney, Tharawal Aboriginal Corporation and the Aboriginal Medical Service Redfern. | **Study design:** A semi-structured qualitative approach was used in focus groups and small group interviews (n 47) to elicit participants’ views on the appropriateness of the SDQ and any additional issues of importance to Aboriginal child and adolescent mental health. | **Outcome measure:** Appropriateness of the SDQ  **Measurement/screening tool:** The Strengths and Difficulties Questionnaire (SDQ).  **Reported reliability/validity with study population**:  The SDQ was found to cover many important aspects of Aboriginal child and adolescent mental health, however, the wording of some questions was considered ambiguous and some critical issues are not explored. The peer relationships subscale did not appear to fit well with Aboriginal concepts of the relative importance of different interpersonal relationships. | **Outcomes:** Overall the SDQ was acceptable in ACCHOs in Sydney; however, changes to the wording of some questions and the response scale may be indicated to improve cultural appropriateness and clarity. A further set of issues which are not covered by any commonly used screening tools but are of critical importance to Aboriginal child and adolescent mental health should also be considered by clinicians. |
| **Williamson 2014 [34]** | **Study aims**: explores the construct validity of the standard Strengths and Difficulties Questionnaire (SDQ) for Aboriginal children living in urban communities in New South Wales, Australia | **Country:** Australia  **Target group** Aboriginal children aged 4–17 years who participated in the baseline survey of the Study of Environment on Aboriginal Resilience and Child Health | **Sample size:** : 717 Aboriginal children aged 4–17 years  **Study setting:** Four Aboriginal Community controlled health services located in urban and large regional centres in New South Wales | **Study design:** SEARCH is the largest cohort study of urban Aboriginal children ever conducted. Participants consent to follow up and to have their information linked to routinely collected population health data. As part of the baseline survey, questionnaire data are obtained from parents/ caregivers and children, along with signed permission for follow-up through repeat data collection and data linkage. All children have their height, weight, waist circumference, and blood pressure measured as well as complete audiometry, otoscopy/pneumatic otoscopy, and tympanometry. Children aged 1–7 years have their speech and language assessed, and their parents/caregivers complete the Parental Evaluation of Developmental Status. | **Outcome measure:**  **Measurement/screening tool:**  **Reported reliability/validity with study population**: overall construct validity of the SDQ in our sample was acceptable but not “good.” The internal consistency reliability was excellent overall and good for all subscales with the exception of peer problems. The convergent validity of the SDQ was good. The prosocial behaviours scale and both the peer and conduct problems scales were highly correlated suggesting Aboriginal parents conceptualise these differently and that prosocial behaviours may be considered a key indicator of well-being for Aboriginal children. | **Outcomes:** Overall, the SDQ is a promising tool for urban Aboriginal children in New South Wales. Those working with Aboriginal young people should focus on the SDQ total difficulties score and limit their reliance on the peer relationships subscale. |
| **Williamson (2016) [35]**  **Journal article** | **Study aims**: To identify the factors associated with ‘good’ mental health among Aboriginal children living in urban communities in New South Wales, Australia. | **Country:** Australia  **Target group**: Aboriginal children and adolescents aged 4–17 years | **Sample size:** 1005 Aboriginal children aged 4–17 years who participated in phase I of the Study of Environment on Aboriginal Resilience and Child Health (SEARCH).  **Study setting:** Four Aboriginal Community Controlled Health Services that deliver primary care. | **Study design:** Cross-sectional survey  The parents or carers of participating children aged 4–17 years completed the standard Australian SDQ about their child by interview with an Aboriginal research assistant. | **Outcome measure:** Carer report version of the Strengths and Difficulties Questionnaire.  **Measurement/screening tool:** The Strengths and Difficulties Questionnaire (SDQ) - Questionnaire content is based on the Western Australian Aboriginal Child Health Survey and the New South Wales Child Health Survey, where possible. Carers completed a survey about their children outlining their physical health, nutritional intake, exercise habits and development. Carers also completed a questionnaire about themselves covering a range of demographic, social, lifestyle and health-related factors, including health service use, com- munity and family resilience and quality and safety of housing.  The Kessler-10 (K10) scale was used to measure psychological distress in carers. A score of 22 or higher was used to indicate high levels of psychological distress consistent with previous literature.  **Reported reliability/validity with study population**: The SDQ has previously been found to be acceptable,[^21^](https://bmjopen.bmj.com/content/6/7/e011182#ref-21) reliable and valid[^22^](https://bmjopen.bmj.com/content/6/7/e011182#ref-22) among the SEARCH cohort. The Kessler-10 has been found to be valid for Aboriginal adults in New South Wales | **Outcomes:** The majority (72%) of SEARCH participants were not at high risk for emotional or behavioural problems. After adjusting for the relative contributions of significant demographic, child and carer health factors, the factors associated with good mental health among SEARCH children were having a carer who was not highly psychologically distressed (OR=2.8, 95% CI 1.6 to 5.1); not suffering from frequent chest, gastrointestinal or skin infections (OR=2.8, 95% CI 1.8 to 4.3); and eating two or more servings of vegetables per day (OR=2.1, 95% CI 1.2 to 3.8). Being raised by a foster carer (OR=0.2, 95% CI 0.01 to 0.71) and having lived in 4 or more homes since birth (OR=0.62, 95% CI 0.39 to 1.0) were associated with significantly lower odds of good mental health. Slightly different patterns of results were noted for adolescents than younger children. |
| **Williamson, et al (2018) [28]** | **Study aims:** The aim of the Study of Environment on Aboriginal Resilience and Child Health (SEARCH) study is to quantify mental health-related emergency department (ED) presentations and hospitalisations, and associated child and family characteristics, in children recruited through four Aboriginal Community Controlled Health Organisations. | **Country:** Australia  **Target group:** Aboriginal children and adolescents in New South Wales**:** The mean age at recruitment into the study was 6.73 years; the median age was 5.86 years. | **Sample size:** 1476 Aboriginal children aged 0–17 years at recruitment to the Study of Environment on Aboriginal Resilience and Child Health.  **Study setting:** Four Aboriginal Community Controlled Health Services that deliver primary care. All services were located in urban or large regional centres in New South Wales, Australia. An ACCHS is defined as ‘a primary health care service initiated and operated by the local Aboriginal community to deliver holistic, comprehensive and cultur- ally appropriate health care to the community that controls it (through a locally elected board of management)’. | **Study design:** Prospective cohort study including linkage to population health data. | **Outcome measure:** ED presentations and hospital admissions with a primary mental health diagnosis. Carers completed a questionnaire based on the Western Australian Aboriginal Child Health Survey and the New South Wales Child Health Survey, including the Australian Strengths and Difficulties Questionnaire (SDQ) as an indicator of level of risk for emotional or behavioural problems.  **Measurement/screening:** ED presentations and hospital admissions with a primary mental health diagnosis obtained via linkage to population health datasets  **Reported reliability/validity with study population** International Statistical Classification of Diseases and Related Health Problems (ICD-10 AM) codes used to identify mental health-related presentations and admissions /hospital stays with a mental health-related principal diagnosis code recorded for the initial episode of care. The SDQ has previously been found to be acceptable, reliable and valid among the SEARCH cohort. | **Outcomes:** Data indicated that 28% of participants aged 4–17 years were at a high risk of clinically significant emotional or behavioural problems, compared with 7.6% of all children in New South Wales (NSW). Over a median of 6-year follow-up, there were 96 ED presentations affecting 62 children (10.7/1000 person-years) and 49 hospitalisations affecting 34 children (5.5/1000 person-years) for mental health conditions. Presentations/admissions increased with age. ED presentation was increased with: living in foster versus parental care (adjusted rate ratio (RR)=3.97, 95% CrI 1.26 to 11.80); high versus low baseline child emotional/ behavioural problems (adjusted RR=2.93, 95% CrI 1.50 to 6.10); and caregiver chronic health conditions versus none (adjusted RR=2.81, 95% CrI 1.31 to 6.63). Hospitalisations were significantly increased with caregiver unemployment versus home duties (adjusted RR=4.48, 95% CrI 1.26 to 17.94) and caregiver chronic health problems versus none (adjusted RR=3.83, 95% CrI 1.33 to 12.12). There were no deaths recorded during the follow-up period |
| **Young (2013) [19]**  **Journal article** | **Study aims**: To identify concepts of health and well-being important to Aboriginal children and youth. These concepts were used to develop a culturally appropriate measure of health. | **Country:** Canada  **Target group**: Aboriginal children and youth between the ages of 8 and 18 years. | **Sample size:** 38 children and youth. The participants ranged in age from 8.2 to 17.7 years (mean age=12.3).  **Study setting:** The Wikwemikong Unceded Indian Reserve Health Centre’s Medicine Lodge and other community settings. | **Study design:** Mixed methods. The research process included community consultation sessions, advisory committee meetings, focus groups. The focus groups engaged Aboriginal children and youth via relevant cultural teachings, a photography exercise combined with a community bicycling tour, and detailed discussions of health and well-being using photovoice. The participants placed their photos on a wall mural and identified their most important concepts. These concepts were synthesized through expert consensus into items and reviewed by the broader community. | **Outcome measure:** Concepts of health and well-being important to Aboriginal children and youth.  **Measurement/screening tool:** The research process resulted in the development of the Aboriginal Children’s Health and Well-Being Measure (ACHWM).  **Reported reliability/validity with study population**: Reported later in following study | **Outcomes:** Through innovative methods, children and youth identified 206 concepts representing the 4 quadrants of the Medicine Wheel: emotional, spiritual, physical and mental. These concepts were refocused, in collaboration with the community, to create a new 60-item measure of health and well-being that was primarily positive in focus, the Aboriginal Children’s Health and Well-Being Measure (ACHWM). |
| **Young (2015)**  **[20]**  **Journal article** | **Study aims**: To assess the validity of the Aboriginal Children’s Health and Well-Being Measure (ACHWM), an Aboriginal developed, culturally appropriate measure of child health and wellbeing. | **Country:** Canada  **Target group**: First Nations children and youth between the ages of 7 to 19 years from one First Nation reserve. | **Sample size:** 48 participants.  **Study setting:** The Wikwemikong Unceded Indian Reserve Health Centre’s Medicine Lodge and other community settings. | **Study design:**  Participants were asked to complete the ACHWM independently using a computer tablet. Participants also completed the Pediatric Quality of Life Scale (PedsQL). | **Outcome measure:** The ACHWM total score and 4 Quadrant scores were validated against the PedsQL total score, domains (scale scores), and summary scores.  **Screening/measurement tools:** Aboriginal Children’s Health and Well-Being Measure (ACHWM)  **Reported reliability/validity with study population**: The Pearson’s correlation between the total ACHWM score and a total PedsQL aggregate score was 0.52 (p = 0.0001). The correlations with the Physical Health Summary Scores and the Psychosocial Health Summary Scores were slightly lower range (r = 0.35 p = 0.016; and r = 0.51 p = 0.0002 respectively) and approached the expected range. The ACHWM Quadrant scores were moderately correlated with the parallel PedsQL domains ranging from r = 0.45 to r = 0.64 (p ≤ 0.001). The Spiritual Quadrant of the ACHWM did not have a parallel domain in the PedsQL. | **Outcomes:** These results establish the validity of the ACHWM. The children gave this measure an Ojibway name, Aaniish Naa Gegii, meaning “how are you?”. This measure is now ready for implementation, and will contribute to a better understanding of the health of Aboriginal children. |
| **Young et al. (2015a) [21]** | **Study aims:** to ensure that children and youth interpreted the ACHWM questions consistently and accurately and to establish the face validity of the survey. | **Country:** Canada  **Target group:** Children and parents/caregivers from the Wikwemikong Unceded Indian Reserve (Canada) participated in a detailed interview process as they completed the ACHWM, in 2012 | **Sample size:** 18 interviews, with 9 children and 9 caregivers  **Study setting:** | **Study design:** Children and parents were interviewed as they completed the ACHWM, working through their thought processes verbally, to enable the interviewer to identify questions that were misinterpreted or inconsistently interpreted. Questions were revised based on feedback from participants, and reviewed with new participants until a stable version was established. The resulting version was reviewed by health care providers and community members to further ensure cultural relevance and face validity. | **Outcome measures:** Establish the face validity of the ACHWM  **Screening/ measurement**  ACHWM  **Reported reliability/ validity with study population** The 58-question version of the ACHWM was consistently interpreted and culturally appropriate, and had face validity confirmed by experts from the community, children and their parents/caregivers. | **Outcomes** Revisions were required for 19 questions. Most of these revisions were minor linguistic changes. In addition, 6 questions were deleted due to consistent problems and 4 questions were created to address gaps identified during the process. Community members confirmed the appropriateness of the measure for their community and communicated their pride in their youth’s role in the development of this survey. The ACHWM is ready to be assessed for relevance to other Aboriginal communities. |
| **Young (2016) [22]**  **Journal article** | **Study aims**: To describe the screening process, embedded within the Aboriginal Children’s Health and Well-Being Measure (ACHWM), and assess its effectiveness. | **Country:** Canada  **Target group**: Aboriginal children and adolescents aged 8 to 18 years of age living on the Wiikwemkoong Unceded Territory. | **Sample Size:** 293 participants between the ages of 8 and 18.  **Study setting:** Participants were recruited from Wiikwemkoong as part of annual community surveys conducted in 2014 and 2015. They were recruited at local schools, the health centre, youth centre, and community events, or as part of an evaluation of the Outdoor Adventure Leadership Experience (OALE) program in 2015. | **Study design:**  Survey responses were screened to identify potential risk, using an automated algorithm run on computer tablets. Local mental health workers conducted brief mental health assessments to identify and support children at-risk. Data were analyzed to estimate effectiveness of this screening process. | **Outcome measures:** The effectiveness of the risk screening process embedded in the ACHWM, and the portion of young people completing the ACHWM being screened as being at-risk.  **Screening/measurement tools:** The Aboriginal Children’s Health and Well-Being Measure (ACHWM).  **Reported reliability/validity with study population**: Created for and with Aboriginal children and youth, between the ages of 8 and 18 years, to assess health from their perspectives. The initial development work was conducted in collaboration with First Nations children in Wiikwemkoong Unceded Territory. | **Outcomes:** A total of 293 children completed the ACHWM. The screening tool identified 35% with potential risk. Mental health workers confirmed 18% of all participants as being at-risk, and all were referred for support. The sensitivity of the tool was 75% while specificity was 79%. Improvements to the screening algorithm resulted in a specificity of 97% and negative predictive value of 95%, with no loss of sensitivity. |
| **Young (2016a)**  **Journal article** | **Study aims:** to evaluate the reliability of the Aboriginal Children’s Health and Well-Being Measure© (ACHWM).[23] | **Country:** Canada  **Target group:** Two cohorts of children from Wiikwemkoong Unceded Territory | **Sample size:** 124 participants in the first cohort and 132 participants in the second cohort  **Study setting:** First Nations children living on the Wiikwemkoong Unceded Territory[^1^](https://www.ncbi.nlm.nih.gov/pmc/articles/PMC5142163/#Fn1) were invited to participate in a program of research that included annual community health surveys in 2014 and 2015, and a repeated measures study that was incorporated into the 2015 community survey. The latter included a sub-set of children who were invited to complete the survey twice. | **Study design:** The internal consistency of the items was assessed using Cronbach’s alpha for each cohort separately, to determine the stability of the estimates. The level of acceptability was specified a priori as 0.7. Data from the test–retest subgroup was assessed using a random-effects Intra-class Correlation Coefficient (ICC) to assess concordance. The ICC values were interpreted as: excellent >0.75, fair to good 0.40–0.75, fair/poor <0.40. | **Outcome measures:**Establish the relaiability of the ACHWM  **Screening/ measurement**  ACHWM  **Reported reliability/ validity with study population:** The internal consistency statistic (Cronbach’s alpha) was 0.93 for the first and second cohorts. The test–retest reliability ICC was 0.94 (95% CI 0.86–0.97) for the ACHWM summary scores based on the repeated measures subgroup. These results establish the internal consistency and the test–retest validity of the ACHWM. | **Outcomes:** results establish the internal consistency and the test–retest validity of the ACHWM and associated quadrant scores. These are important parameters that will enable Aboriginal communities across Canada to use this measure with confidence. Because the measure is based on child-self-report, it promotes the voices of children in understanding their health. The establishment of these key measurement properties is essential to enable evidence-based health care within Aboriginal communities. The ACHWM is now ready for use in evaluating the impact of various programs on Aboriginal children’s health. |
